# RNA Viral Community in Human Feces: Prevalence of Plant Pathogenic Viruses

**DOI:** 10.1371/journal.pbio.0040003

**Published:** 2005-12-20

**Authors:** Tao Zhang, Mya Breitbart, Wah Heng Lee, Jin-Quan Run, Chia Lin Wei, Shirlena Wee Ling Soh, Martin L Hibberd, Edison T Liu, Forest Rohwer, Yijun Ruan

**Affiliations:** **1**Genome Institute of Singapore, Singapore; **2**Department of Biology, San Diego State University, San Diego, California, United States of America; University of North CarolinaUnited States of America

## Abstract

The human gut is known to be a reservoir of a wide variety of microbes, including viruses. Many RNA viruses are known to be associated with gastroenteritis; however, the enteric RNA viral community present in healthy humans has not been described. Here, we present a comparative metagenomic analysis of the RNA viruses found in three fecal samples from two healthy human individuals. For this study, uncultured viruses were concentrated by tangential flow filtration, and viral RNA was extracted and cloned into shotgun viral cDNA libraries for sequencing analysis. The vast majority of the 36,769 viral sequences obtained were similar to plant pathogenic RNA viruses. The most abundant fecal virus in this study was pepper mild mottle virus (PMMV), which was found in high concentrations—up to 10^9^ virions per gram of dry weight fecal matter. PMMV was also detected in 12 (66.7%) of 18 fecal samples collected from healthy individuals on two continents, indicating that this plant virus is prevalent in the human population. A number of pepper-based foods tested positive for PMMV, suggesting dietary origins for this virus. Intriguingly, the fecal PMMV was infectious to host plants, suggesting that humans might act as a vehicle for the dissemination of certain plant viruses.

## Abstract 2

A comparative metagenomic analysis of RNA viruses in the human gut identifies the vast majority as plant pathogens.

## Introduction

The human gastrointestinal tract is the natural habitat for a large microbial community including species from the kingdoms Archaea, Bacteria, and Eukarya [1]. It is estimated that the human gastrointestinal (GI) flora contains 10^14^ microorganisms. Most of these microbes are symbiotic to the human host and beneficial to food digestion [2,3]. The GI microbiota also contains enteric viruses, including a variety of bacteriophages and a number of known human viruses and uncharacterized viruses. Bacteriophages can influence food digestion by regulating microbial communities in the human GI tract through lytic and lysogenic replication [4]. Bacteriophages may also contribute to human health by controlling invading pathogens [5]. In addition to bacteriophages, the other well-studied human enteric viruses are the viral pathogens associated with gastroenteritis. They can infect the human small intestine cells, causing damage to the epithelial lining and the absorptive villi, leading to the malabsorption of water and an electrolyte imbalance [6,7]. Many viral pathogens have been isolated from the feces of gastroenteritis patients, including rotavirus, astrovirus, calicivirus, hepatatis E virus, certain members of coronavirus and torovirus, and the enteric adenovirus (serotypes 40 and 41) [8–11]. Except for the adenoviruses, which contain DNA genomes, all the others are RNA viruses. Despite intensive studies, many causative agents of human gastroenteritis are still unknown.

Traditionally, discovery of viruses was dependent on culturing the viruses in host cells in order to propagate and isolate enough pure virions for characterization. However, it is generally known that the large majority of viruses, including enteric viruses, cannot be cultivated using standard techniques. For instance, some Norwalk viral agents causing gastroenteritis could not be grown in cell cultures [12,13]. The only way to obtain adequate virus particles for characterization of these viruses was to feed the volunteer human adults with stool filtrates derived from the disease outbreak [14]. Hence, culture-based methods are insufficient for large-scale characterization of the viral community in the human GI tract. In addition, viruses do not have ubiquitously conserved genetic elements such as rDNA that can be used as diversity and evolutionary distance markers [15].

Metagenomic analyses offer an opportunity to directly characterize mixed genomes of uncultured viruses. In combination with tangential flow filtration for viral particle isolation and concentration from large volume samples, metagenomic analyses have been employed to survey the DNA viruses in seawater [16] and from human feces [17]. Based on the analysis of about 500 sequences, it was found that the majority of DNA viruses in human feces were novel, and most of the recognizable sequences belonged to bacteriophages. Similar metagenomic approaches have also been applied, in large scale, to bacteria in seawater [18] and other environmental samples [19,20].

To date, very little information is available on the human enteric RNA viral flora, despite the fact that many RNA viruses are known etiologic agents of gastroenteritis. To expand our understanding of the RNA viral flora in the human GI tract, we conducted a comprehensive metagenomic analysis of uncultured RNA viruses isolated from the feces of healthy humans. Surprisingly, we discovered that some plant RNA viruses were highly abundant in human feces.

## Results

### Identification of RNA Viruses in Human Feces

Three fecal samples from two healthy adults living in San Diego were used for virus isolation. Samples 1 and 2 were from the same individual, with a gap of 6 mo between the sample collections. Sample 3 was from a second individual. We concentrated the feces-borne viral particles using tangential flow filtration as described previously [17]. We further treated the viral concentrates with DNase and RNase to eliminate potential contamination with free nucleic acids. Viral RNA was extracted from each sample and converted into cDNA, which was used to construct a shotgun library for sequencing analysis. From the three libraries (designated as Lib 1, Lib 2, and Lib 3 corresponding to samples 1, 2, and 3), we generated 10,576, 13,572, and 12,621 high-quality sequence reads, respectively (Table 1). Each sequence read represents an individual clone in the libraries.

**Table 1:**
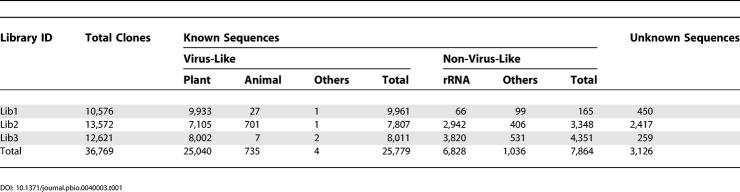
Clone Sequences in Three RNA Viral Shotgun Libraries

Of the total 36,769 sequences, 33,643 (91.5%) were similar to known sequences in the GenBank nr (nonredundant) database based on tBLASTx (e-value <0.001), and are therefore designated as “known” (Table 1). Of these 33,643 known sequences, 25,779 (76.6%) were most similar to viruses and 7,864 (23.4%) were most similar to Bacteria, Archaea, or Eukarya. Of the nonviral known sequences, 6,828 (86.8%) were similar to ribosomal RNA. The presence of ribosomal RNA-like sequences in these libraries may reflect the copurification of some ribosomes with the virions from the fecal extracts during tangential flow filtration.

Based on the best hit of tBLASTx analysis, the virus-like sequences were assigned to 42 viral species, including two animal viruses, 35 plant viruses, one yeast virus, and four bacteriophages (Table 2). The number of sequences related to yeast viruses and bacteriophages in these libraries were insignificant, with only one and seven clones matching them, respectively. Surprisingly, of the virus-like sequences, 25,040 (97.1%) were homologous to plant viruses, while less than 735 (2.9%) resembled animal viruses. This is distinct from the previously reported DNA fecal viral library, in which most virus-like sequences were similar to phages [17]. Furthermore, no RNA phage-like sequences were observed in this study, which may reflect the rare occurrence of RNA phages in human feces [21]. Notably, very few DNA virus-like sequences (eight clones) were found in these libraries, indicating that DNA contamination was essentially eliminated during our isolation procedure.

**Table 2:**
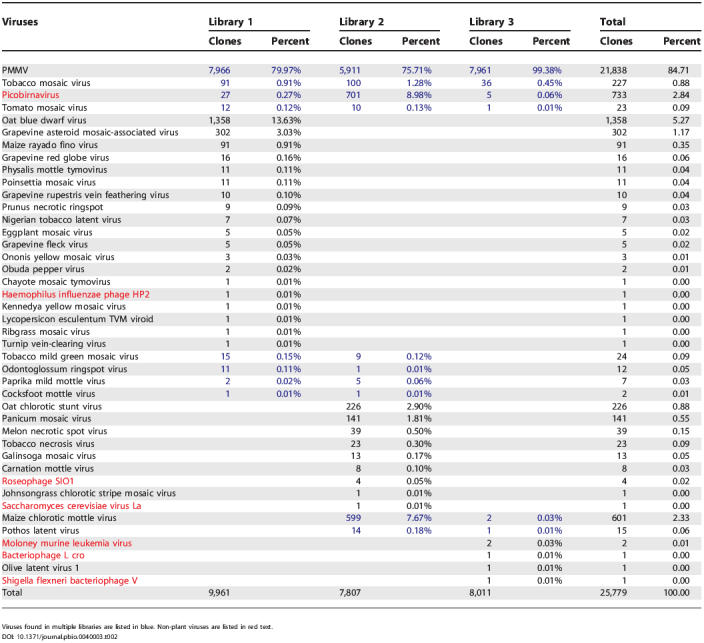
Viruses Identified in Three RNA Viral Libraries

The most abundant virus in the fecal RNA viral libraries was pepper mild mottle virus (PMMV). PMMV is a well-characterized plant pathogenic virus that infects all species in the genus *Capsicum,* including a wide variety of sweet and hot peppers [22,23]. A total of 21,838 sequences were mapped across the entire genome of PMMV (6,357 bp). The levels of sequence homology to the PMMV reference genome sequence were highly variable, suggesting that multiple variants of PMMV might be present in these samples. In addition, 24 out of the 35 plant viruses detected in these fecal samples were known pathogens of consumable crops including fruits, vegetables, tobaccos, and cereals (Table 3), suggesting that foods contaminated with plant viruses might have contributed to the RNA viral flora in human feces.

**Table 3:**
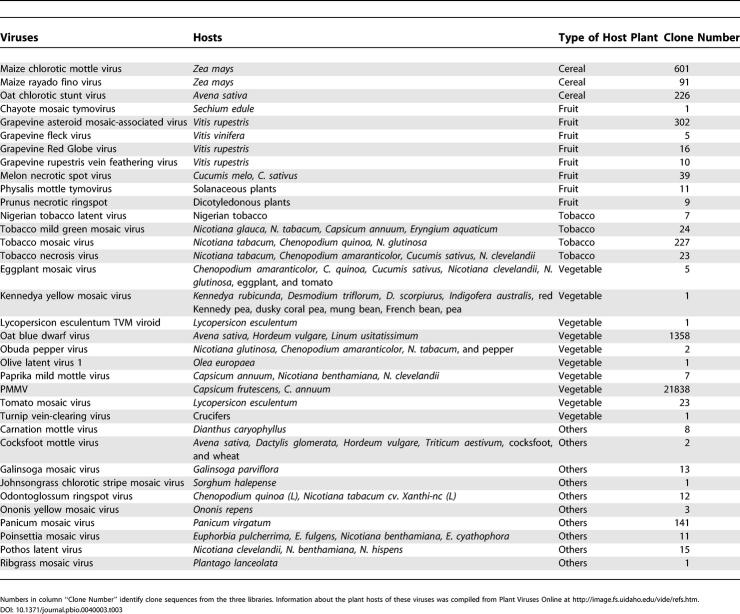
Plant Viruses Identified in Human Feces and Their Known Host Plants

The most common animal virus (total of 735 clones) identified in the RNA viral libraries was a picobirnavirus (PBV, 733 clones). PBV belongs to a recently identified family of RNA viruses with bisegmented genomes, which are present in the stools of humans and animals [24,25]. In humans, PBV has been found in both healthy individuals and diarrhea patients [26]. The other animal virus identified (Moloney murine leukemia virus) was only represented by two clones. In summary, we found that the human feces-borne RNA viral community is dominated by viruses similar to plant RNA viruses, exemplified by the abundance of PMMV.

### The Diversity and Dynamics of the RNA Viral Community in Human Feces

Of the 42 viral species identified, 32 were present in only one of the libraries. Among the common viral species, four were present in all three libraries, and six were found in only two libraries. Hence, the overlap between these libraries was quite small in terms of the types of viral species (see Table 2).

Although there were 27, 24, and ten viral species identified in Lib 1, 2, and 3, respectively, the majority of the clones in these libraries were represented by only a few dominant viral species (see Table 2). For instance, in Lib 1, PMMV, oat blue dwarf virus, and grapevine asteroid mosaic-associated virus were the top three species represented by 80%, 13.6%, and 3.0% of the 9,961 virus-like clones, respectively. In Lib 2, PMMV (75.7%), PBV (8%), maize chlorotic mottle virus (MCMV, 7.7%), oat chlorotic stunt virus (OCSV, 2.9%), panicum mosaic virus (PanMV, 1.8%), and tobacco mosaic virus (1.3%) were the six major viral species of the 7,807 virus-like clones. In Lib 3, PMMV was the only dominant viral species, accounting for 99.4% of the 8,011 virus-like sequences. It is interesting to note that, except for PMMV, which was predominant in all three libraries, the other major species found in each of the three libraries were quite different. For instance, oat blue dwarf virus and grapevine asteroid mosaic-associated virus were the second and third most abundant viruses in Lib 1, but were undetectable in Lib 2. Similarly, MCMV, OCSV, and PanMV were the second, third, and fourth most abundant viruses in Lib 2, but were not found in Lib 1. Since Lib 1 and Lib 2 were derived from feces collected from the same individual 6 mo apart, these differences most likely reflect fluctuations in the fecal RNA viral community of a single individual over time.

To further address the dynamics of human feces-borne viruses, we compared the PBV-like clone sequences from Lib 1 and Lib 2 (Figure 1). It is known that human PBV has two distinct genogroups, represented by two prototype strains, 4-GA-91 and 1-CHN-97 [26,27]. The members of the former genogroup are highly conserved, while the members of the latter are quite variable. The sequence identity between the two prototypes is 28% at the nucleotide level and 40% at the amino acids level. To facilitate the comparative analysis, we clustered the PBV-like sequences of each library into contigs (using minimum overlaps of 40 bp and over 95% identity) and aligned them to the partial genome sequences of the 4-GA-91 and 1-CHN-97 available in GenBank. The five contigs from 27 PBV-like sequences from Lib 1 were highly similar (over 90% identical at nucleotide level) to the conserved PBV strain 4-GA-91 (Figure 1A) but had no matches to the other prototype strain. In contrast, the 26 contigs from 701 PBV-like sequences from Lib 2 were only marginally homologous (46%–69% homology at the amino acid level) to the variable strain 1-CHN-97 (Figure 1B). It is important to note that the PBV populations in the two fecal samples (samples 1 and 2) from the same individual represented distinct genogroups, once again highlighting the dynamic nature of the human enteric RNA viral flora.

**Figure 1:**
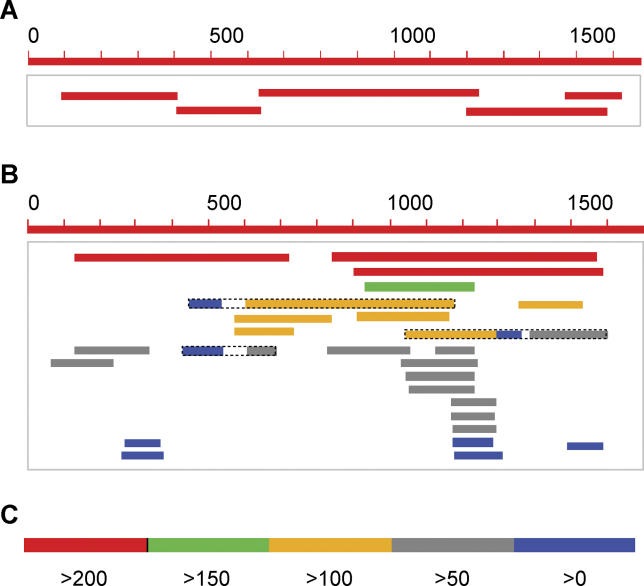
Different PBV Strains Found in Two Fecal Samples from the Same Individual (A) The PBV-like sequence segments identified in Lib 1 were aligned to the partial genome sequence of PBV strain 4-GA-91 using BLASTn. The identities of nucleotide sequence between the contigs and the reference PBV sequence were 95%–99%. (B) The PBV-like sequence segments in Lib 2 were too remote to both known PBV strains (4-GA-91 and 1-CHN-97) at the nucleotide level, but could be aligned to the PBV strain 1-CHN-97 using tBLASTx. The identity of amino acid sequences between the PBV-like sequence segments in Lib 2 and the reference PBV genome sequence were 46%–69%. (C) Colored bars indicate the similarity level between library sequences with template sequences as measured by BLAST score.

Similarly, the other fecal viruses, including PMMV, identified in this study also appeared variable. Using a minimum overlap of 40 bp and more than 95% identity, we clustered all PMMV-like sequences and generated 376 (Lib 1), 359 (Lib 2), and 225 (Lib 3) unique PMMV genome sequence segments. The largest PMMV contig was 3,158 bp in length and contained 1,817 clones. Even though the feces-borne PMMV sequences gave over 200-fold coverage of the reference PMMV genome, a complete fecal PMMV genome could still not be assembled de novo. As shown in Figure 2, the PMMV-like sequences within each library had a wide range of BLAST scores to the PMMV reference genome, suggesting that the feces-borne PMMV sequences were very divergent and represented novel PMMV variants. Similar results were observed for other viral species (unpublished data).

**Figure 2:**
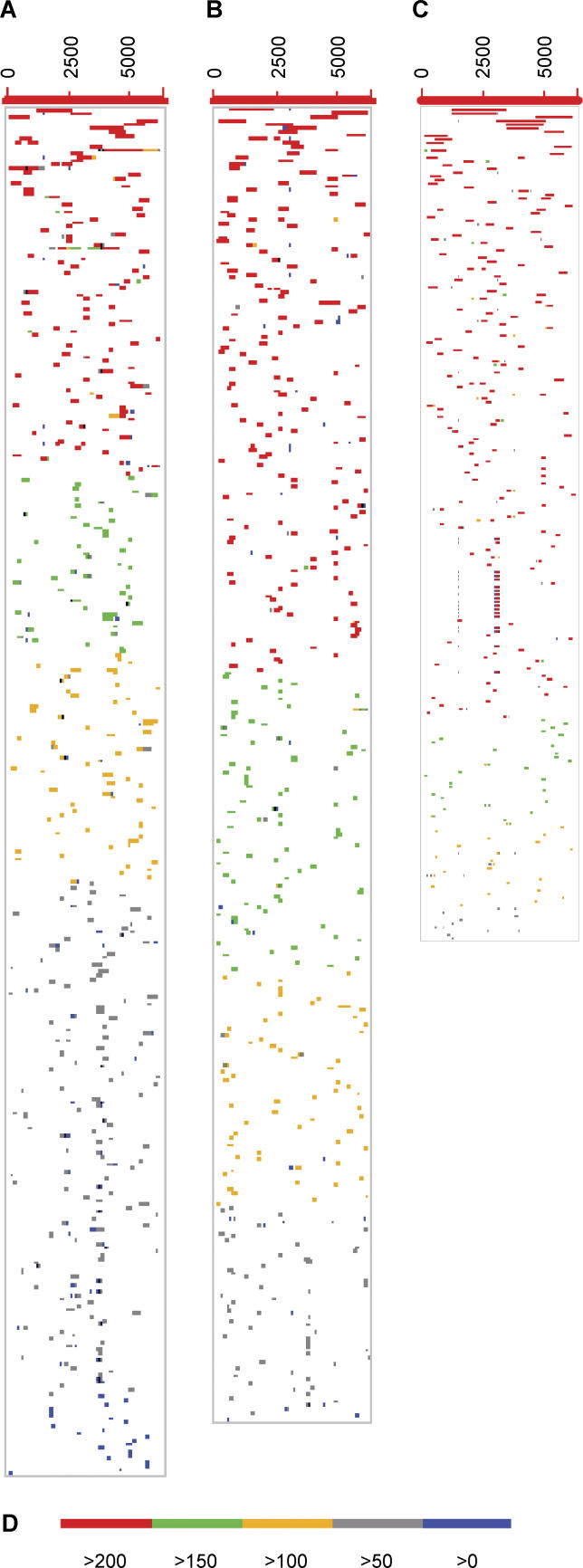
Alignment of Assembled PMMV-Like Sequences from Three Shotgun Libraries with the Reference PMMV Genome Sequence The PMMV-like viral genome sequence segments from Lib 1 (A), Lib 2 (B), and Lib 3 (C) were aligned with the reference PMMV genome sequence (6,357 bp). Colored bars (D) indicated the similarity level between library sequences with template sequences as measured by BLAST score.

We further analyzed the phylogenetic relationship of PMMV variations in these libraries. There are seven complete PMMV genomes and 14 individual PMMV coat protein (CP) gene sequences available in GenBank. To utilize the maximum number of reference sequences, we decided to use the CP gene for phylogenetic analysis together with the PMMV-like sequences identified in the three libraries. Since the first 101 bp of the 474-bp CP gene (from 5,685 to 6,158 bp of the PMMV genome) had the most sequences (44 unique assembled PMMV sequence segments) mapping to it, we chose this region for phylogenetic analysis. The majority of the 44 feces-borne PMMV sequences were grouped into five main phylogenetic clusters (Figure 3). The previously known PMMV CP gene sequences were found mostly in three clusters (clusters I, II, and III). Interestingly, most of the PMMV sequences found in Lib 1 were grouped in cluster IV, however, only one sequence from Lib 2 was grouped into this cluster, indicating that the PMMV strains were very different even in two fecal samples collected from the same individual, and implying that the human fecal PMMV population was dynamic.

**Figure 3:**
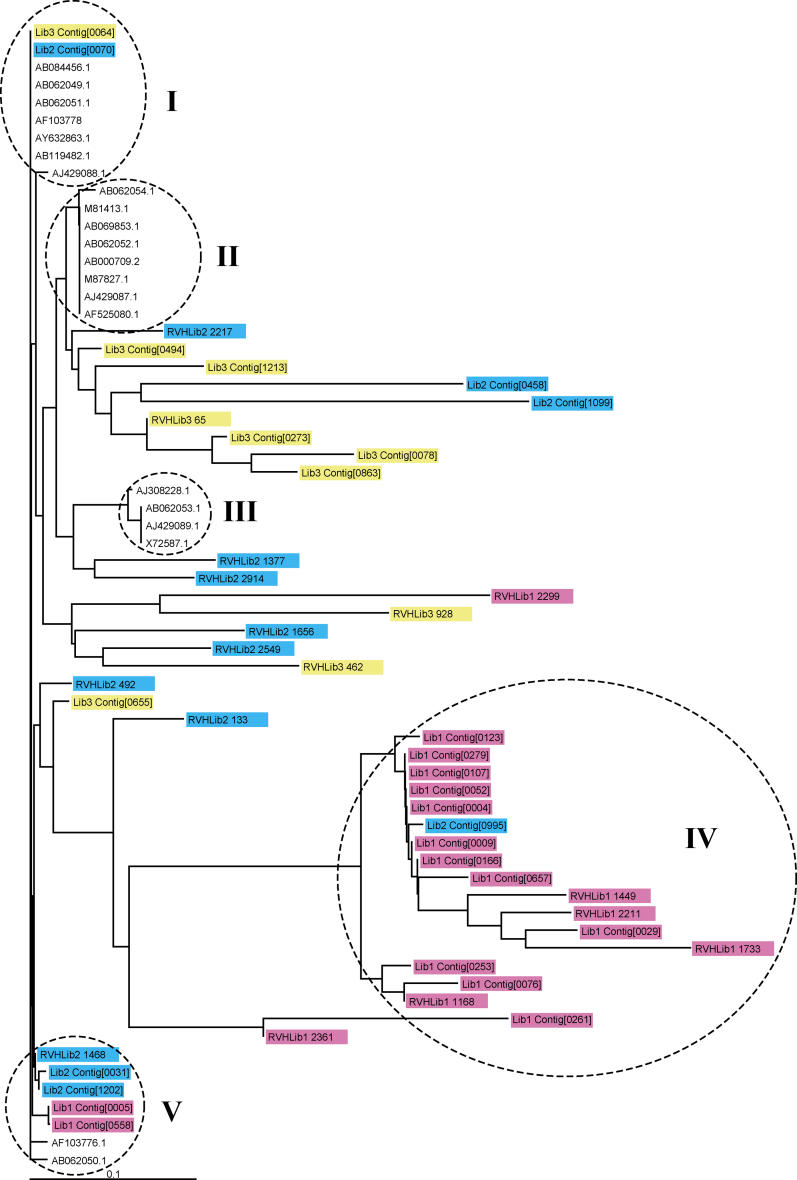
Phylogenetic Tree of PMMV Sequences A region of 101 bp in the PMMV CP gene was chosen for sequence comparison and phylogenetic analysis. A total of 44 assembled sequences (>50 bp) from the three fecal virus libraries were located within this region. These sequences were aligned with 21 known PMMV CP genes from GenBank using ClustalW with default parameter settings. In this phylogenetic tree of the PMMV CP gene, sequences from Lib 1 are highlighted in pink, Lib 2 in blue, and Lib 3 in yellow. GenBank accession numbers of known CP genes are shown unshaded.

Taken together, our sequence analysis clearly showed that the RNA viral flora in human feces is diverse and dynamic at both species and strain levels, although we cannot rule out the possibility that the heterogeneous nature of human feces and different sequencing coverage in these libraries may also contribute to the variations observed.

### The Prevalence of PMMV in the Human Population

To confirm the presence of the viruses identified through shotgun library sequencing, we used RT-PCR to detect these viruses directly from the fecal samples used for library construction. We first detected the presence of PMMV in a fecal sample and validated that our RT-PCR detection is specific and reliable (Figure 4A and 4B). We then extended the test for the five most abundant viruses identified in Lib 2 (PMMV, MCMV, PBV, OCSV, and PanMV; see Table 2), and all were positive from the primary fecal sample 2 (Figure 4C). Furthermore, the relative quantities of the RT-PCR products for each of the viruses tested were in good correlation with the relative abundance reflected by the number of clones observed for each of the viruses in the shotgun library.

**Figure 4:**
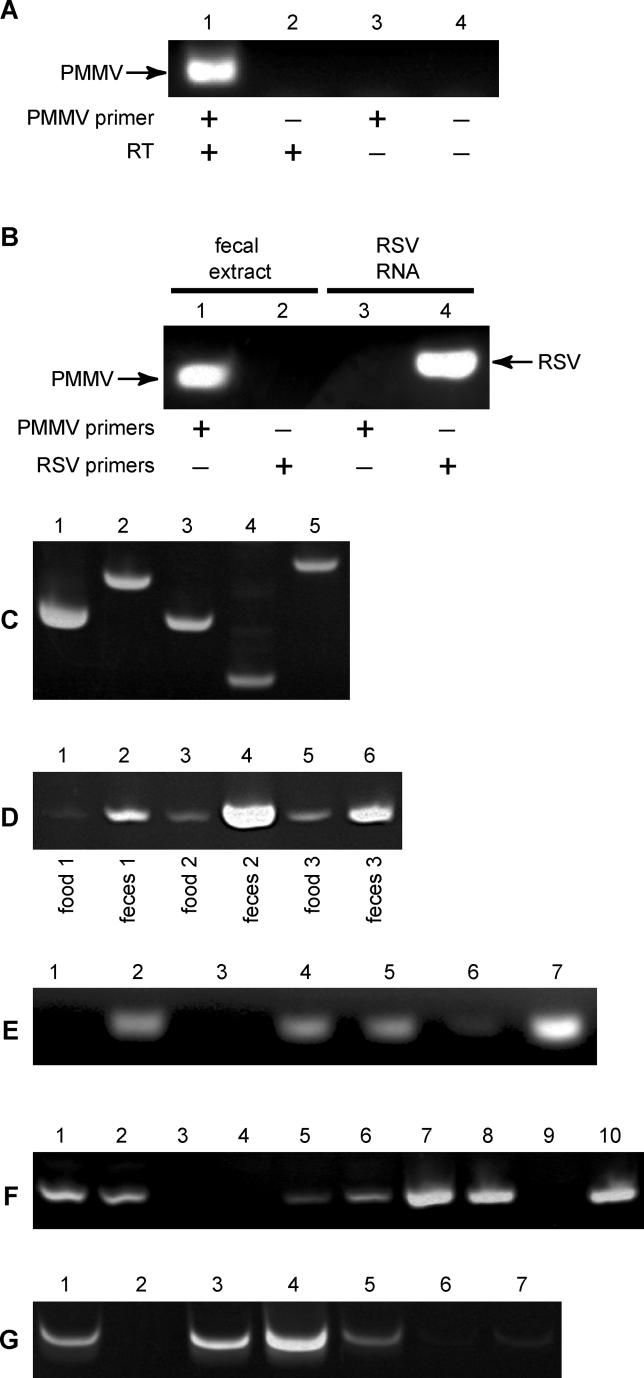
RT-PCR Detection of Fecally Borne RNA Viruses (A) PMMV (lane 1) was detected by RT-PCR using PMMV specific primers in a fecal RNA extract. This PMMV band is PMMV primer-specific (lane 2) and dependent on reverse transcription (lane 3). (B) The specificity of the RT-PCR reaction for detecting fecal PMMV was further assessed by the use of nonspecific PCR primers (lane 1 versus lane 2) and respiratory syncytial virus (RSV; American Type Culture Collection #VR-1401) as a nonspecific RNA template (lanes 1 and 2 versus lanes 3 and 4). The identities of RT-PCR products (PMMV in lane 1; RSV in lane 4) were confirmed by sequencing analysis. (C) RNA viruses were directly detected by RT-PCR from the total RNA of fecal sample 2: PMMV (lane 1), MCMV (lane 2), PBV, segment 2 (lane 3), OCSV (lane 4), and PanMV (lane 5). (D) Equal amounts of dry weight of food (meal samples for 2 d prior to fecal collection) and feces from three individuals were assayed by RT-PCR to compare the amounts of PMMV present. The estimated numbers of virions in 1 g of dry food and feces were 1.21 × 10^6^ (lane 1), 2.3 × 10^7^ (lane 2), 1.63 × 10^7^ (lane 3), 3.64 × 10^9^ (lane 4), 2.42 × 10^7^ (lane 5), and 1.95 × 10^8^ (lane 6) as determined by TaqMan RT-PCR. (E) Fecal samples from six additional individuals from San Diego were analyzed for detection of PMMV. The positive control is shown in lane 7. (F) Detection of PMMV in fecal samples of nine individuals from Singapore, including one infant (lane 9). Lane 10 is the positive control. (G) Detection of PMMV from seven chili sauces from Singapore.

Since the plant viruses identified from the libraries were mostly food crop pathogens, we suspected that the plant viruses found in feces might have originated in food. We then used PMMV as the model to test the existence of plant viruses in food, and estimated the viral loads in food and feces by TaqMan quantitative RT-PCR assay. Three pairs of food-fecal samples from three healthy individuals were collected for total RNA extraction. For each sample pair, small aliquots were taken from all the foods consumed by the participants for 2 d prior to the time of feces collection. The quantity of RNA used for the RT-PCR assay was adjusted based on equal dry weight. As shown in Figure 4D, PMMV was found in both food and feces from all three individuals, and the virus copy number was in the range of 10^6^–10^9^ per gram of dry feces based on the TaqMan quantitative RT-PCR results. The PMMV viral load in feces was enriched 20-fold (Figure 4D; lane 2 versus 1), 224-fold (lane 4 versus 3), and 8-fold (lane 6 versus 5) in pairwise comparisons with food (estimated virus copy numbers are shown in Figure 4). Besides the possible experimental error in measurement, the differences in enrichment of PMMV in feces might reflect the different levels of reduction and condensation of mass from food to feces through absorption during digestion in different individuals and at different times.

To determine the prevalence of PMMV among human fecal samples, six more fecal samples were collected from different individuals in San Diego, California, United States for RT-PCR analysis. Three of these additional samples were positive for PMMV (Figure 4E; lanes 2, 4, and 5). Together with the previous three positive fecal samples tested (Figure 4D; lanes 2, 4, and 6), the overall positive rate was 6/9 (66.7%) in San Diego. To ascertain if PMMV is found only in North Americans, we tested nine human fecal samples (including one from a newborn) from Singapore, and six were positive for PMMV (Figure 4F; lanes 1, 2, and 5–8). Notably, the infant fecal sample was negative for PMMV (lane 9). Hence, the presence of PMMV in feces is not geographically restricted. Among the nine individuals from Singapore, six had had peppers in their recent meals. Interestingly, PMMV was detected in feces from five of them.

Since peppers were indicated as a potential source of PMMV, we collected a panel of pepper-containing foods from San Diego for RT-PCR testing. Out of 22 fresh and processed pepper samples tested, only three were positive, including bottled hot chili sauce and powdered chili (unpublished data). Interestingly, none of the fresh, healthy-looking peppers tested were positive for PMMV. To further investigate the presence of PMMV in chili sauces, seven randomly collected samples from food stalls in Singapore were tested; four of these chili sauces contained PMMV (Figure 4G; lanes 1 and 3–5). Together, these data demonstrate that food is a potential source of PMMV in human feces, and that intact PMMV nucleic acids can survive standard food processing.

We then assessed if the feces-borne PMMV viruses were viable and able to infect host plants. Two Hungarian wax pepper *(Capsicum annuum)* plants were inoculated with a small amount of PMMV-positive fecal suspension. Within 1 wk, all inoculated plant leaves developed typical symptoms of PMMV infection (Figure 5), while the five control plants remained healthy. Total RNA extracted from the infected and healthy leaves of the same plant were tested for PMMV (Figure 5B). PMMV could only be detected in the infected leaf (Figure 5B; lane 2), and this PCR diagnostic band was confirmed by sequencing. Thus, the PMMV shed from the human GI tract is still infectious to host plants.

**Figure 5:**
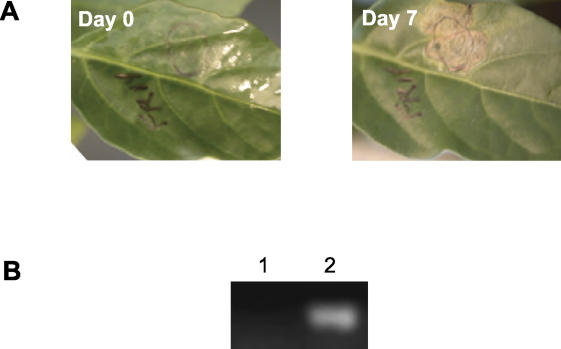
Fecally Borne PMMV Is Infectious to a Pepper Plant (A) Fecal supernatant containing PMMV was inoculated on to a leaf of a *Capsicum* plant (Day 0). After 7 d of inoculation, this leaf developed typical symptoms of viral infection (Day 7). (B) RNA extracts from uninfected control leaves (lane 1) and PMMV-positive fecal supernatant challenged leaves (lane 2) were tested for PMMV by RT-PCR.

## Discussion

The original intention of this study was to identify human enteric viruses or other human viruses through a metagenomic survey of uncultured RNA viruses in human feces. However, we found that animal viruses were uncommon in healthy human feces and were limited to PBVs. It might be possible that human viruses replicating in mucosal cells were undetectable in stools from healthy individuals but are abundantly shed into feces in inflammatory conditions when symptoms such as diarrhea develop [28]. Therefore, analysis of fecal samples from diarrhea patients would be the logical extension of this study to identify unknown pathogenic human enteric viruses.

Unexpectedly, we discovered a large and diverse community of plant RNA viruses exemplified by PMMV in human feces. PMMV was found in two-thirds of the individuals tested from two different continents (San Diego in North America and Singapore in Southeast Asia), suggesting that PMMV is prevalent in human populations. We also observed that the RNA viral community in human feces was dynamic. The representation of RNA viruses found in a single individual over time or in different individuals varied substantially. Considering that most of the feces-borne plant viruses identified in this study are known to be pathogenic to vegetable crops and that PMMV was detectable in processed pepper food (chili sauces), we speculate that foods might be the major source of fecally borne RNA viruses. Fluctuations of the RNA viral populations in human feces may be associated with the kinds of food consumed, methods of food preparation, and idiosyncratic conditions in the GI tract.

We further provide evidence that the fecal PMMV was viable and could cause infection to a host plant. PMMV viruses appeared to be stable through the human digestive system. This is consistent with the observation that members of *Tobamovirus,* including PMMV, are extremely robust viruses and can survive in challenging environments [29,30]. The data presented here suggest that humans (and likely other animals) may play a significant role as transmission vectors for certain plant viruses through their digestive tracts. The discovery of this previously unknown transmission route for plant viruses is potentially important for a better understanding of certain agricultural practices, because in traditional agriculture and in developing countries, human and animal refuse are often used as fertilizers for crops.

Since the PMMV was abundant in the fluid phase of fecal material by RT-PCR and library sequencing, it is conceivable that these viruses might disassociate from the plant tissues and therefore are readily accessible to host epithelial cells and microbial cells existing in the intestinal environment. Earlier studies showed that plant viral particles could be assembled in *Escherichia coli* cells [31,32], which leads to the speculation that some feces-borne plant viruses might be capable of interacting with microbes in human guts. In this study, the amount of fecal PMMV appeared to be significantly higher than the virus load found in food based on equal dry weight. This increase could be a result of the digestive reduction of food, or viral replication in the human gut. PMMV, like other members of *Tobamovirus,* is a plus-stranded RNA virus [33]. During replication in host cells, a complementary minus-strand RNA is synthesized from the genomic plus-stranded RNA template and serves as the template for genomic RNA. Hence, evidence of viral replication is the presence of the minus-strand RNA. We were unable to detect the minus strand of PMMV as an indication of replication using a strand-specific RT-PCR method [34,35]. Hence, the evidence for active replication of PMMV in human feces is currently lacking. Further research efforts are needed to determine whether and how plant viruses may interact with intestinal cells or microorganisms in the human GI tract.

This study demonstrates the existence of abundant and dynamic populations of live plant pathogenic viruses in human feces. It is already known that complex symbiotic interactions exist between viruses, plant hosts, and their insect vectors [36,37]. For the tomato yellow leaf curl virus, the lifespan of its whitefly vector, *Bemisua tabaci,* is reduced by one-fifth, and their fecundity is reduced by one-half during viral transmission [38], which suggests that viral replication may affect health of the vector. Given that nonpathogenic commensal intestinal bacteria can significantly alter the immune system of an organism [39], it is conceivable that viruses such as PMMV, replicating or not, may also have an effect. Nevertheless, the stability of PMMV, and perhaps other plant viruses, in the human GI tract may allow them to be used as a platform for oral vaccine development [40] to prevent diarrhea and other intestinal disorders.

## Materials and Methods

### Fecal samples

Three fecal samples from two healthy adults living in San Diego, California, United States were collected for RNA viral isolation and sequencing analysis. Samples 1 and 2 were from the same individual with a gap of 6 mo between sample collections. Sample 3 was from a second individual. The two human participants in this study had no history or recent symptoms of diarrhea or any known gastrointestinal illness, and had a reasonably balanced daily diet of meat, vegetables, and grains (bread, rice, or noodle).

Additional fecal samples were obtained later for RT-PCR detection of specific feces-borne viruses. Six fecal samples were collected from six healthy humans who worked together in the same building, but lived in different locations in the San Diego area. Subsequently, nine fecal samples were collected from nine individuals living in Singapore (including one infant). For the Singapore group, we undertook a comprehensive record and analysis of food intake prior to sample donation (Table 4). The food intake of participants was documented over a period of 3 d prior to fecal sample collection. Foods were grouped by their main nutritional composition, namely protein, carbohydrate, fat, dietary fiber, and calcium. Foods that contain added microorganisms were categorized as probiotics.

**Table 4:**
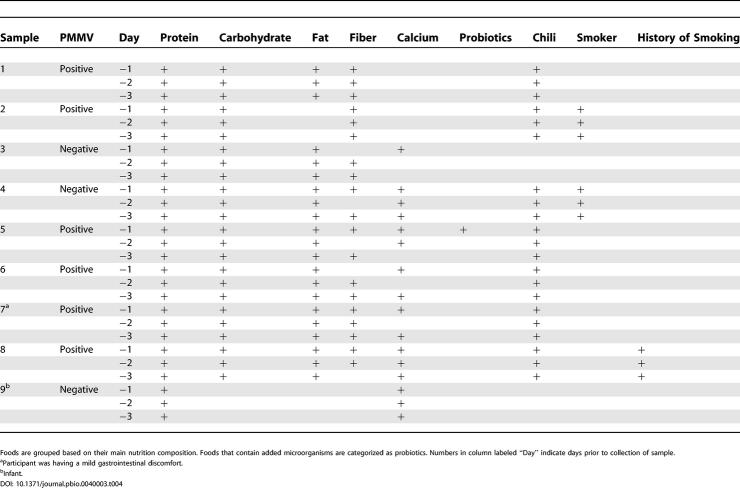
Food Intake of Singapore Participants Three Days Prior to Donating Sample

### Viral isolation

Viral isolation and concentration were performed as described previously [17]. Briefly, approximately 500 g of fresh fecal matter was resuspended in 5 l of SM buffer (0.1 M NaCl, 50 mM Tris-HCl [pH 7.4], 10 mM MgSO_4_), and this material was passed through a Nitex filter (pore size ~100 μm) followed by filtration through a 0.2-μm tangential flow filter (Amersham Biosciences, Little Chalfont, United Kingdom). The viral particles were then concentrated on a 100-kDa tangential flow filter (Amersham Biosciences). The viral concentrates were stained with SYBR Gold (Invitrogen, Carlsbad, California, United States) and visualized using epifluorescent microscopy to ensure the recovery of viruses and removal of microbial cells [41]. DNase I and RNase A were added to a final concentration of 1 unit per 100 μl of viral concentrate, and the sample was incubated at 37 °C for 3 h to remove any contamination by free nucleic acids. Viral RNA was extracted using the Qiagen RNA/DNA Midi Kit (Qiagen, Valencia, California, United States) according to the manufacturer's instructions, and was then treated with DNase to eliminate any DNA contamination.

### Viral RNA shotgun library construction

Approximately 140–200 ng of purified viral RNA of each sample was utilized for cDNA synthesis using a random priming approach following the protocol from SuperScript Choice System (Invitrogen). Double-stranded cDNA was divided into two aliquots, which were digested separately with either TaqI (New England Biolabs, Beverly, Massachusetts, United States) or Csp6I (Fermentas, Burlington, Ontario, Canada) to generate short overlapping DNA fragments. TaqI- and Csp6I-digested cDNA fragments were ligated with TaqI and Csp6I adaptors (oligo sequences in Table 5) at 16 °C overnight, respectively. The TaqI and Csp6I adaptor-ligated DNA fragments were amplified by PCR using forward primers TaqI-F and Csp6I-F for 40 cycles, respectively. DNA fragments between 500 and 1,000 bp were fractionated and cloned into Zero Blunt TOPO PCR cloning vectors (Invitrogen) for sequencing.

**Table 5:**
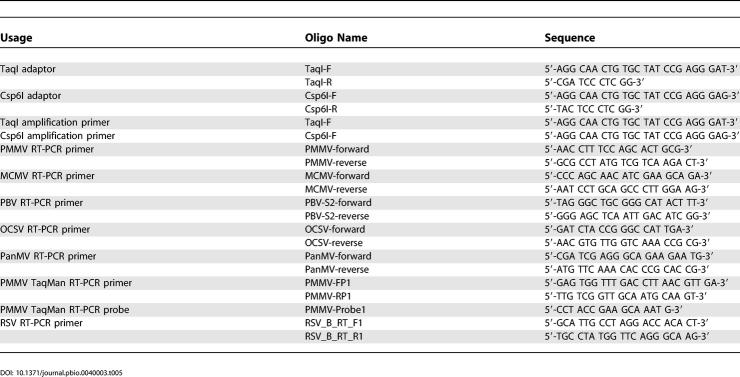
Oligonucleotide Sequences of Adaptors, Primers, and Probes Used in This Study

### Sequence production and analysis

The viral cDNA clones were prepared as plasmid templates for sequencing by an ABI3730 DNA analyzer (Applied Biosystems, Foster City, California, United States). The raw sequence reads were subjected to base calling and vector/adaptor trimming using PHRED [42,43]. High-quality sequences for all three libraries were searched for homology against the nonredundant nucleotide database (nr) in GenBank using tBLASTx [44,45]. Sequences that had tBLASTx hits with an e-value less than 0.001 were considered known, and the top hit was assigned to the particular sequence for annotation; otherwise they were considered unknown. Based on the best tBLASTx hit, virus-like sequences were further classified based on hosts, such as plant, animal, or bacterial origins. Nonviral sequences were subjected to BLASTn against the European ribosomal RNA database (http://www.psb.ugent.be/rRNA/index.html). Sequences that had BLAST hits to this database with an e-value less than 0.001 were considered to be structural ribosomal RNA.

### Sequence clustering

For each library, shotgun viral sequences were clustered into longer viral genome segments. We first used PHRAP [46] with highly stringent parameters (<qual_show 20>, <confirm_length 50>, <confirm_score 100>, <minscore 180>, and <maxgap 0>), and generated 2,553 unique viral genome sequence segments that included 257 contigs and 2,296 singletons from Lib 1, 4,958 (708 contigs and 4,250 singletons) from Lib 2, and 3,546 (405 contigs and 3,141 singletons) from Lib 3. The PHRAP-generated unique viral sequence segments were further clustered using the Sequencher program with the parameters of a minimum of 40 bp overlap and greater than 95% nucleotide identity, which resulted in 1,109 (297 contigs and 812 singletons), 1,746 (409 contigs and 1,337 singletons), and 1,184 (270 contigs and 914 singletons) unique viral sequence segments for Lib 1, 2, and 3, respectively.

### Phylogenetic analysis of PMMV sequences

The PMMV CP gene was used for phylogenetic analysis. There are 21 known PMMV CP gene sequences available in GenBank. From the three libraries, 44 assembled fecal PMMV sequence segments were aligned within the first 101 bp of the 474 bp CP gene (from 5,685 to 6,158 bp of the PMMV genome). ClustalW (http://www.ebi.ac.uk/clustalw/) was used for phylogenetic comparison of these sequences, and TreeView (Win32) was used to generate the phylogram tree (http://taxonomy.zoology.gla.ac.uk/rod.html).

### RT-PCR detection of fecally borne RNA viruses

Approximately 20 g of each fecal sample was diluted 1:1 with phosphate-buffered saline and homogenized for 1 min at medium speed in a Stomacher 400 (Seward, Thetford, Norfolk, UK) [47,48]. The samples were then centrifuged at 10,000 *g* for 5 min to sediment particulate matter, and the supernatant was subjected to nucleic acid extraction using the Qiagen viral RNA Mini kit. Approximately 100 ng of fecal RNA was used for first strand cDNA synthesis using virus-specific reverse primers (Table 5). The reverse transcription reaction was performed using SuperScript II RNase H-reverse transcriptase following the manufacturer's protocol (Invitrogen). The RT products (1/10 of total reaction) were used as templates for PCR reactions using virus-specific primer pairs (Table 5) and amplifying for 35 cycles. PCR products were analyzed by electrophoresis on agarose gels. The identities of all PCR products were confirmed by sequencing. All RT-PCR tests included RNA-less and RT-less samples as negative controls.

Food samples analyzed in this study were subjected to the same procedure for RNA extraction and RT-PCR detection of PMMV.

### TaqMan assay for quantification of PMMV

To determine the copy number of PMMV in fecal and food samples, a TaqMan assay was used to quantify the viral load in these samples according to the manufacturer's recommendation (Applied Biosystems). Briefly, the assay was performed in a 20-μl reaction mixture containing 2 μl of fecal RNA, five units of MuLV reverse transcriptase, eight units of recombinant RNase inhibitor, 10 μl of 2× universal PCR Master Mix with no UNG (Applied Biosystems), 0.9 μM primers (PMMV-FP1 and PMMV-RP1; Table 5), and 0.2 μM probe (PMMV-Probe1; Table 5). The real-time RT-PCR reactions were carried out in an ABI Prism 7900HT Sequence Detection System (Applied Biosystems). The RT reaction was performed at 48 °C for 30 min, followed by 10 min at 95 °C for activation of DNA polymerase, and then 40 cycles of 15 s at 95 °C and 1 min at 60 °C. Negative controls (RT-less and RNA-less) and serial dilutions of positive control (a plasmid clone) were included in every PCR assay.

### Infection of plants with fecally borne viruses

Two PMMV-positive fecal samples (10 g) were resuspended in 10 ml of SM buffer, centrifuged at 12,000 *g* for 15 min to pellet the bacteria and large debris, and the supernatants were mixed with ground glass. Two healthy Hungarian wax pepper *(Capsicum annuum)* plants were used for the test, and two leaves per plant were inoculated with 2 μl of the sample supernatant/ground glass mixture with gentle grinding followed by rinsing with sterile water. The inoculated leaves were then monitored visually over a period of 2 wk, and photos were taken with a digital camera to document viral infection symptoms. The leaves that developed viral symptoms were collected for RNA extraction and viral detection by RT-PCR and subsequent sequencing analysis. Negative controls of five plants were subjected to the same inoculation procedure, with the exception of the fecal supernatant addition.

## Supporting Information

### Accession Numbers

The GenBank (http://www.ncbi.nlm.nih.gov/) accession numbers of organisms discussed in this paper are PMMV genome (NC_003630) and PBV prototype strains 4-GA-91 (AF246948) and 1-CHN-97 (AF246939).

## Abbreviations

CP: coat protein
GI: gastrointestinal
MCMV: maize chlorotic mottle virus
OCSV: oat chlorotic stunt virus
PanMV: panicum mosaic virus
PBV: picobirnavirus
PMMV: pepper mild mottle virus
RT: reverse transcription
